# 3D Peri-Implant Epi-Mucosa-on-a-Chip Reveals Alterations in Epithelial Barrier Function Mediated by Host-Bacteria-Biomaterial Interactions

**DOI:** 10.1021/acsbiomaterials.5c01232

**Published:** 2025-11-12

**Authors:** Sana Surrency, Soraya Tarrah, Mashael Al Thuanayan, Yoontae Kim, Rahul Patil, Alison Grafton, Micaila Curtis, Peter Lialios, Georgios A. Kotsakis, Stella Alimperti

**Affiliations:** † Department of Biochemistry and Molecular & Cellular Biology, 8368 Georgetown University, Washington, DC 20057, United States; ‡ Center for Biological and Biomedical Engineering, 8368 Georgetown University, Washington, DC 20057, United States; § King Abdullah bin Abdulaziz University Hospital, Riyadh 11564, Saudi Arabia; ∥ Department of Oral Biology, 212493 Rutgers School of Dental Medicine, Newark, New Jersey 07103, United States; ⊥ Institute for Soft Matter Synthesis and Metrology, 8368 Georgetown University, Washington, DC 20057, United States

**Keywords:** peri-implantitis, barrier function, epithelium, titanium, shear stress, bacteria, microfluidics

## Abstract

Peri-implantitis is characterized by disruption of the epithelial barrier at the implant–mucosa interface, driven by complex interactions between mechanical, microbial, and material factors. Histological and immunohistochemical analysis of human peri-implant and periodontal tissues revealed significant epithelial abnormalities specific to peri-implantitis, which indicated compromised barrier integrity. Specifically, peri-implant tissues had increased intercellular edema, inflammatory infiltration, and marked loss of junctional proteins E-cadherin and ZO-1. To further investigate these findings in a controlled environment, we developed a novel *3D Peri-implant Epi-mucosa-on-a-chip* model incorporating clinically relevant titanium surfaces, hydrostatic pressure, and bacterial challenge to mimic peri-implant crevicular fluid dynamics and disease pathogenesis. Using this microfluidic platform, we demonstrated that untreated titanium surfaces significantly increased epithelial leakiness and disrupted the localization of junctional proteins, such as E-cadherin. In contrast, acid-etched titanium with defined microroughness restored barrier function and preserved junctional integrity. High hydrostatic pressure, mimicking inflammatory mechanical stress, independently impaired epithelial cohesion, whereas the combination of *Porphyromonas gingivalis* (*P. gingivalis*) and implant-derived titanium microparticles (i-TiPs) synergistically exacerbated barrier breakdown. i-TiPs also altered matrix architecture and stiffness, further compromising epithelial integrity and potentiating bacterial damage. These results underscore the critical role of implant surface properties and the mechanical microenvironment in modulating host barrier responses and highlight the utility of our 3D model in elucidating the mechanobiology of peri-implantitis. This platform may pave the way for the development of new therapeutic strategies against peri-implantitis, aimed at preserving epithelial sealing and preventing disease progression.

## Graphical abstract

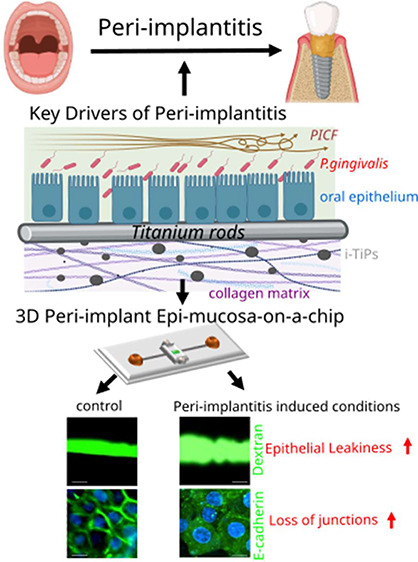

## 1 Introduction

Dental implants are an indispensable part of restoring the quality of life of people who experience tooth loss. However, they also pose the risk of prevalent, destructive inflammatory conditions, named peri-implant diseases,^1^ which lead to patient-perceived morbidity. Healthy peri-implant mucosa has a similar appearance to healthy gingiva with no visual signs of inflammation, such as erythema (rubor) or edema (tumor). During disease initiation, incipient disease is restricted to the peri-implant soft tissues, i.e., peri-implant mucositis, and the removal of microbial biofilm from the implant provides reversibility by eliminating the etiology. While peri-implant diseases begin with biofilm formation, emerging evidence implicates compromised epithelial sealing as a critical initiating event and barrier failure as a pathognomonic hallmark.^2–4^ The pathognomonic clinical sign of peri-implant diseases is bleeding on probing (BOP), which is assessed as bleeding when the peri-implant sulcus is gently probed.^5–7^ When inflammatory diseases progress to the underlying peri-implant bone, i.e., peri-implantitis, current antimicrobial treatments, such as antibiotics, or surgical decontamination approaches, do not yield long-term effectiveness.^8–10^ Therefore, early intervention by developing new treatments is key to preventing disease progression, preserving implant longevity, and ensuring patients’ overall satisfaction and quality of life.

The epithelium lines the sulcus and forms a seal around the implant, providing an important barrier against bacteria and their byproducts.^11–13^ Despite titanium implants being biocompatible, the biomaterial substrate may contribute to epithelial damage by promoting bacterial adhesion and biofilm formation in the sulcus microenvironment, which induces inflammation, loss of epithelial barrier function, and further bone degradation.^14,15^ In disease, peri-implant bacteria such as *Porphyromonas gingivalis* (*P. gingivalis*) increase in abundance, forming complex biofilms on titanium and implant surfaces.^16–18^ In addition to affecting host immune activation, these bacteria also affect the titanium biomaterial by causing microbial biocorrosion, which significantly increases microtitanium particle release.^19^ Multiple human studies have consistently reported that implant-derived Titanium Particles (i-TiPs) are significantly increased in the peri-implant microenvironment in cases of peri-implantitis compared to healthy conditions.^20–29^ We have previously shown that these i-TiPs, which are from biocorrosion-abrasive damage to the titanium passivation layer during dental cleaning procedures,^30^ are found in high concentrations around diseased implants, whereas their concentrations are near zero around healthy implants.^30,31^ The biological significance of these i-TiPs, which act as abiotic exposomes that amplify the inflammatory cascade, is evident by the refractory nature of peri-implantitis to antimicrobial and antibacterial interventions.^32^ In addition to the impact of microtitanium particle and bacteria in peri-implantitis, a critical mediator of this inflammatory process is the peri-implant crevicular fluid (PICF), which accumulates in the peri-implant sulcus.^33,34^ During peri-implant inflammation, the volume of PICF increases due to vascular leakiness and immune cell infiltration, creating a pro-inflammatory environment that supports the growth of pathogens.^17,33–37^ Beyond this inflammation, this fluid buildup creates high hydrostatic pressure in the peri-implant sulcus, which can biomechanically impact the epithelial function.^34,37^

To further elucidate the mechanisms driving peri-implant epithelial barrier breakdown and the progression of peri-implant diseases, it is critical to develop advanced in vitro models that recapitulate both the mechanical and biological complexity of the peri-implant environment. Toward this goal, we have developed a three-dimensional (3D) microfluidic model, named the *3D Peri-implant Epi-mucosa-on-a-chip*. This model was designed to capture the complex host-microbiome-biomaterial interactions that govern peri-implant homeostasis. Specifically, this platform enables a systematic investigation of how key pathological factors, including the PICF, peri-implant pathogens (e.g., *P. gingivalis*), and i-TiPs, interact with the epithelium and modulate barrier integrity. Specifically, we used this system to assess the impact of these stimuli on epithelial barrier function and the expression of key junctional proteins, such as E-cadherin and ZO-1, which are essential for the regulation of the epithelial peri-implant seal. Overall, this approach provides a biomimetic platform to dissect the molecular and biomechanical mechanisms underlying peri-implantitis and to identify potential therapeutic targets to preserve peri-implant tissue health.

## 2 Materials and Methods

### 2.1 Cell Culture

Human Immortalized Gingival Keratinocytes (HIGKs)^38,39^ were maintained in Keratinocyte-Serum Free Media (SFM) (Thermo Fisher Scientific, USA), supplemented with prequalified human recombinant Epidermal Growth Factor 1–53 (EGF 1–53) and Bovine Pituitary Extract (BPE; Thermo Fisher Scientific, USA). All experiments were conducted using HIGKs at passages 7–9.

### 2.2 SEM Analysis of Titanium Rods and i-TiPs Particles

Titanium rods (diameter 1 mm) (Wen-Titanium, China) were treated with 0.2% v/v Hydrofluoric Acid (HF) in a Teflon dish for 120 s. Rods were immediately washed with deionized water and air-dried for 20 min in a fume hood.^40^ The generation of clinically relevant i-TiPs has been described previously.^21^ The surface morphology of the untreated, HF-treated titanium rod, and i-TiPs embedded in a collagen I matrix was observed under Scanning Electron Microscopy (SEM; Zeiss, USA) in high-vacuum mode at magnifications of 2000× and 10,000×, with an accelerating voltage of 30 kV.

### 2.3 Characterization of Titanium Rod Roughness

The roughness of the untreated and HF-treated titanium rods was measured with Atomic Force Microscopy (AFM; NT-MDT, Russia/USA). Surface topography was evaluated under ambient air, in tapping mode, at a scanning rate of 0.2 Hz, and over a 5 × 5 μm^2^ scanning area. Specifically, AFM was conducted using the NTEGRA Prima system (NT-MDT, Russia/USA). Root mean square roughness (RMS) and arithmetic average roughness (Ra) values were calculated using NT-MDT Image Analysis P9 software.

### 2.4 Viscoelastic Characterization

To characterize the viscoelastic properties of the matrix with embedded i-TiPs, shear modulus measurements were performed on a stress-controlled rheometer with a thermal enclosure (MCR302, Anton Paar, Austria), equipped with a 25 mm parallel-plate measuring system and a 1 mm gap height. Approximately 750 μL of type I collagen solution (3 mg/mL, derived from rat tail, Corning, USA) with and without i-TiPs (10^4^ particles/mL, based on vivo data^22^) was loaded onto the rheometer stage at 37 °C immediately after bringing the pH to 7.0. After setting the gap, excess material at the edges was trimmed, and water was added around the sample area to minimize evaporation and control humidity before sealing the thermal enclosure. Measurements were conducted at a constant temperature of 37 °C, with small-amplitude oscillations (5%, well within the linear viscoelastic range) recorded every 10 s over a 25 min period (150 total data points per run). Storage modulus (*G*′), loss modulus (*G*″), and corresponding raw stress–strain signals were monitored. The complex shear modulus was defined as *G*(ω) = *G*′(ω) + *iG*″(ω)**, and the magnitude |*G*| = √(*G*′^2^ + *G*″^2^ *)* at ω = 1 rad/s.

### 2.5 Bacteria Culture

*Porphyromonas gingivalis* (*P. gingivalis*; ATCC 33277) was cultured on Trypticase Soy Agar II plates with 5% w/v sheep blood (MOLTOX, USA) for 7 d to allow colony growth. The plates were inverted and placed in a 2.5 L anaerobiosis jar (Sigma) with an anaerobic gas generator (AnaeroGen, UK) to create an oxygen-depleted environment, then incubated at 37 °C with 5% v/v CO_2_. Anaerotest strips (Sigma, USA) were included in the jar to confirm the presence of an anaerobic atmosphere. Once colonies had formed, individual colonies were picked with a disposable inoculating loop and cultured in microcentrifuge tubes containing Brain Heart Infusion (BHI) media supplemented with hemin (1 mg/mL) and menadione (1 mg/mL; Sigma-Aldrich, USA). The tubes were incubated anaerobically at 37 °C for 20–24 h until the optical density (OD) reached 0.1–0.3. OD was measured using an ND-1000 spectrophotometer (Thermo Fisher Scientific, USA) at 600 nm. The corresponding bacteria dose was calculated based on the equation: cfu/mL = (number of colonies × dilution factor)/(volume plated in mL),^41,42^ yielding an estimated ∼1.25 × 10^8^ colony-forming units (CFU) per mL of *P. gingivalis*, corresponding to an OD_6__0__0_ of 0.1. To achieve a multiplicity of infection (MOI) of 50, 3 × 10^6^ bacteria were added to the device, which contained an estimated 60,000 cells based on the seeding density used to generate the microepithelial tube. Cells were exposed to *P. gingivalis* at an MOI of 50 for 15 min under aerobic conditions. Although *P. gingivalis* is an obligate anaerobe, it demonstrates significant aerotolerance, allowing a subset of the bacteria to survive short periods (i.e., 15 min) of oxygen exposure.^43–45^

### 2.6 Fabrication of Microfluidic Platform

Molds for microfluidic devices were fabricated using a stereolithography (SLA) 3D printer (Form 3+; Formlabs). Computer-aided design (CAD) models were first created and exported as standard triangle language (.STL) files, which were then printed with clear resin (GPCL04; Formlabs) to produce the molds. The uncured resin was removed from the surface of the 3D-printed molds by soaking and moving them in isopropyl alcohol (IPA) using an automated washing system (Form Wash, Formlabs, USA). Next, the washed molds were postcured at 405 nm light by using a multidirectional LED curing chamber (Form Cure, Formlabs, USA) at 45 °C for 45 min. Next, the cured molds were plasma treated for 5 min and salinized overnight in trichloro­(1*H*,1*H*,2*H*,2*H*-perfluorooctyl) silane (Sigma, USA). Polydimethylsiloxane (PDMS; Sylgard 184, Dow-Corning, USA) devices were fabricated from these surface-modified molds. The PDMS devices were treated with 0.01% v/v poly-l-lysine (PLL; Sigma, USA) and 0.5% v/v glutaraldehyde (Sigma, USA) to promote collagen adhesion. After washing overnight in distilled water, the untreated or HF-treated titanium rods were introduced into the PDMS devices. Next, a steel acupuncture needle (diameter = 160 μm Seirin, Japan) was introduced into the PDMS device, followed by the introduction of type I collagen (3 mg/mL), which was subsequently polymerized for 1 h at 37 °C, as described previously.^38^ For experiments involving i-TiPs, type I collagen (3 mg/mL) was mixed with i-TiPs (10^4^ particles/mL) and polymerized under identical conditions for 1 h at 37 °C. The following day, the acupuncture needles were removed to create 160 μm-diameter channels within a polymerized collagen gel, with both sides of the channel attached to collagen (control), one side to an untreated titanium rod, or one side to an HF-treated titanium rod.

Next, a suspension of 10^6^ cells/mL HIGKs was introduced into the PDMS devices. The cells were adhered to the top surface of the channel for 5 min, then flipped to allow them to adhere to the bottom surface for another 5 min. The nonadherent cells were washed out, and fresh media was replaced with the device. To apply mechanical stress, devices were placed on a pressure controller system for 24 h, applying hydrostatic pressure of 0.1 kPa (Low stress) and 10 kPa (High Stress), as described previously.^38,46^ Finally, under those conditions, the cells in the devices were exposed to *P. gingivalis* (MOI = 50) for 15 min, as described in Figure S1.

### 2.7 Epithelial Permeability Measurement

To assess epithelial permeability on the microfluidic platform, fluorescent dextran (40 kDa, Thermo Fisher Scientific, USA) was added to the perfusion media (SFM) at a concentration of 12.5 μg/mL. The diffusion of the dextran was captured in real time using a confocal microscope (LSM 800, Carl Zeiss, Germany) at 10× magnification. A sequence of images was analyzed by calculating the mean intensity over regions adjacent to the cell layer in successive frames. The time derivative of the intensity was obtained through linear regression for each region. The time derivative of intensity, mean intensity (d*I*/d*t*), and capillary radius (*r*) were used to calculate the diffusive permeability coefficient (*P*_d_) using the equation *P*_d_ = (d*I*/d*t*) × (*r*/2*I*). The uncertainty was determined by the standard deviation of the measured permeability at various locations, as previously described.^38,46^

### 2.8 Immunofluorescence Staining and Quantitative Analysis

The 3D microfluidic devices were fixed in 4% v/v paraformaldehyde (PFA; Sigma, USA) for 20 min at 37 °C, washed twice in PBS, permeabilized with 0.1% v/v Triton X 100 in PBS for 3 h at room temperature (RT), and treated with blocking solution (0.01% v/v Triton X 100, 5% w/v goat serum (Sigma, USA) in PBS) overnight at 4 °C. To visualize cell–cell interactions and junctional protein localization, HIGK cells were incubated overnight at 4 °C with E-cadherin (4A2) Mouse mAb (1:100, Cell Signaling, USA) and ZO-1 (40–2200) Rabbit pAb (1:100, Thermo Fisher Scientific, USA). The following day, cells were washed with PBS for 4 h at RT, then incubated overnight at 4 °C with secondary antibodies including 4′,6-Diamidino-2-Phenylindole (DAPI) (1:1000, Sigma), goat anti-Mouse IgG (H + L) Alexa Fluor 488 (1:100, Thermo Fisher Scientific, USA), and goat anti-Rabbit IgG (H + L) Alexa Fluor 568 (1:100, Thermo Fisher Scientific, USA). Next, the cells were incubated with DAPI (1:1000) and Alexa Fluor 555 Phalloidin (1:500, Thermo Fisher) for 1 h at room temperature. Devices were imaged using a confocal microscope (CSU-W1 SD SoRa, Nikon Japan), and image analysis was performed in ImageJ (NIH, USA)^47^ by generating a maximum-intensity z-projection and merging the channels. Image background was cleared using the masking function in ImageJ to enhance visualization of the region of interest while preserving the integrity of the quantitative data. More than 80 cells were measured in three separate experiments. Image analysis was performed using Junctional Mapper software, as reported previously.^48^

### 2.9 Human Studies

Tissue samples were used from the IRB-approved Periodontal Tissue Repository at the University of Texas Health Sciences Center at San Antonio (UTHSCSA) (IRB #: HSC20190310H). Tissue samples were collected from surgically discarded tissues from dental patients undergoing the following procedures. In the peri-implantitis disease group (*N* = 6), soft tissue was collected from explanted failing implants. In the implant health group (*N* = 3), samples were collected during tissue punch procedures during the second-stage surgery. During this procedure, implants are submerged under epithelial tissues, thus free of bacterial interactions on their surfaces and representing absolute tissue health. Lastly, control sites from the periodontal health group (*N* = 3) were collected around healthy teeth during crown lengthening procedures. After tissue collection, samples were placed in 10% formalin overnight and then stored in 70% ethanol (EtOH) for epitope preservation until trimming and processing. Lastly, tissues were embedded in paraffin and sectioned for immunohistochemical staining using an automatic microtome. In addition to preserved tissue sections, cytologic smears were collected using a cytobrush (Brush & Bond, S286, USA) for the assessment of the presence of titanium microparticles in the peri-implant epithelia, followed by staining in Pa.

### 2.10 Immunohistochemical Analysis

Sections were deparaffinized and washed with xylene (Fisher Chemical, USA), followed by dehydration in a graded alcohol series. For H&E staining, the sections were stained with Mayer’s hematoxylin and eosin (H&E; ab245880, Abcam, UK). For immunohistochemical analysis, after dehydration, antigen retrieval was performed (10 mM sodium citrate, pH 6.0; 40 mL per 50 mL tube), followed by blocking with 2.5% horse serum for 30 min at RT. Cells were incubated with the primary antibodies at 4 °C overnight. Then, sections were washed with PBS and incubated with ImmPRESS reagent (Vector Laboratories, Inc.) for 30 min, then mounted on coverslips with 100% glycerol.

### 2.11 Statistical Analysis

Statistical analysis of the data was performed using one-way analysis of variance (ANOVA) with Tukey’s posthoc tests for pairwise comparisons. The *p*-values <0.05 were considered to be statistically significant unless stated otherwise in the text. All results were expressed as mean plus or minus one standard deviation. In each test, the number of independent experiments (*N*) was ≥3, and the number of data points in each experiment was different. Both *N* and *n* are shown in the figure legends. Human specimens were collected from three groups. The first group (*N* = 6) included subjects undergoing peri-implantitis surgery (diseased tissue), the second group (*N* = 3) included subjects undergoing second-stage implant surgery (tissue representing absolute health without bacterial presence), and the third group (*N* = 3) included subjects undergoing nonimplant periodontal procedures around erupted teeth (healthy periodontal tissue). Staining was scored using the scoring system described by Klen et al., where 0 = no staining; 1 = weak; 2 = mild; 3 = strong staining.^49^

## 3 Results

### 3.1 Loss of E-Cadherin and ZO-1 Correlates with Epithelial Barrier Breakdown in Peri-Implantitis

To assess epithelial barrier alterations in peri-implantitis, we conducted human tissue studies comparing peri-implant to healthy periodontal tissues. Histological analysis revealed normal rete ridges and parakeratinized oral epithelium in the periodontally healthy group, with no evidence of nuclear alterations or fragmentation. Vascular and inflammatory infiltrates were present at the junctional epithelium. In the implant health group (second stage implant surgery for tissue removal under submerged implants), the parakeratinized oral epithelium showed slight intercellular edema, without vascular or dense inflammatory infiltration, and nuclear features remained unaltered across all layers. In contrast, the peri-implantitis group exhibited abnormal rete ridges, severe intercellular edema, and dense inflammatory infiltrates in both the junctional and sulcular epithelia, with especially prominent vascular inflammatory infiltration in the sulcular epithelium (Figure 1a).

**Figure 1:**
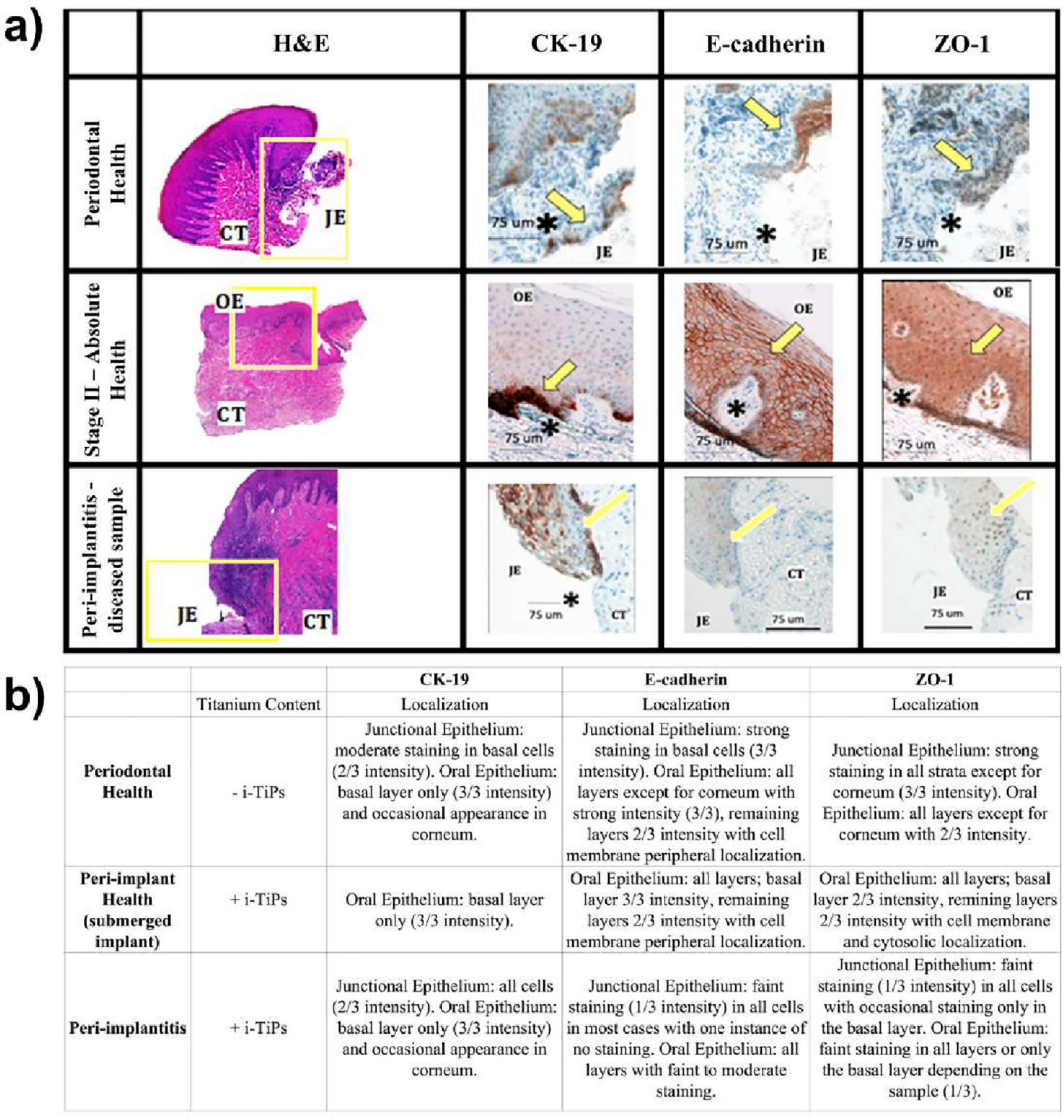
Loss of E-cadherin and ZO-1 correlates with epithelial barrier breakdown in peri-implantitis. (a) Representative H&E and immunohistochemical staining of CK-19, E-cadherin, and ZO-1 show epithelial structure and junctional protein localization in periodontal health, absolute health, and peri-implantitis tissues. Yellow boxed areas in H&E images show the junctional epithelium (JE), while arrows and asterisks indicate notable features such as rete ridge architecture, intercellular edema, and differences in staining intensity across epithelial layers (scale bar: 75 μm). (b) Qualitative assessment of staining intensity and localization for CK-19, E-cadherin, and ZO-1. Titanium presence (+ i-TiPs or – i-TiPs) is indicated for each condition.

Nevertheless, cytologic smears invariably showed metal-like particles in the peri-implantitis (diseased tissue) group, indicating the presence of titanium in this group, whereas the healthy tissue groups were negative for metal particles (Figure S2). Next, we examined the expression of key epithelial markers involved in intercellular junctions—specifically ZO-1 and E-cadherin—and epithelial cell turnover (CK-19), which is a primary form of periodontal tissue defense against bacterial-driven inflammation. In the implant health group, CK-19 was strongly localized to the stratum basale of the oral epithelium, with no junctional epithelium present (Figure 1a). E-cadherin was robustly expressed at cell–cell junctions in the basal and suprabasal layers, with strong staining throughout all epithelial layers (Figure 1b). ZO-1 was detected at the cell periphery from the basal layer to the uppermost living layers of the oral epithelium (Figure 1a). In the periodontal health group, CK-19 showed strong staining in the basal cells of the oral epithelium (Figure 1b). E-cadherin was expressed at the basal and suprabasal junctions of the junctional epithelium and demonstrated strong staining across all layers of both the oral and junctional epithelium. ZO-1 showed mild expression in the junctional epithelium (Figure 1b). In the peri-implantitis diseased tissue group, CK-19 staining showed strong expression across all junctional epithelial layers (Figure 1b). Conversely, E-cadherin expression in the junctional epithelium was generally weak or absent across most samples, with only one showing substantial staining (Figure 1b). ZO-1 localization was reduced, with weak peripheral expression from the basal to the uppermost layers of the junctional epithelium (Figure 1a). Overall, these findings indicate that peri-implantitis is associated with significant epithelial barrier disruption, characterized by structural abnormalities, increased edema, increased epithelial tissue turnover, and diminished expression of key intercellular junction proteins, particularly in the junctional and sulcular epithelia.

### 3.2 Engineering 3D Peri-Implant Epi-Mucosa on a Chip Platform

To leverage the human tissue findings, we aimed to investigate and deconvolute how key contributing factors in peri-implantitis—namely, titanium roughness, PICF, bacteria, and i-TiPs—alter epithelial functionality in a translational peri-implant model (Figure 2a). To this end, we developed a novel 3D microfluidic platform termed the *3D Peri-implant Epi-mucosa-on-a-chip* (Figure S1). This device was engineered using a 3D-printed scaffold by casting a hollow cylindrical channel (160 μm in diameter) into a collagen I matrix integrated with titanium rods. The setup was embedded within a PDMS mold with a central bulk chamber to support the channel and reservoir chambers for media introduction (Figure 2b,c). Epithelial cells were seeded within the channel and organized into a structured microepithelial tube, as shown in Figure 2d. Barrier integrity was evaluated using real-time microscopy by tracking the diffusion of 40 kDa dextran, as illustrated in Supplementary Video S1 and supported by prior studies.^38^

**Figure 2:**
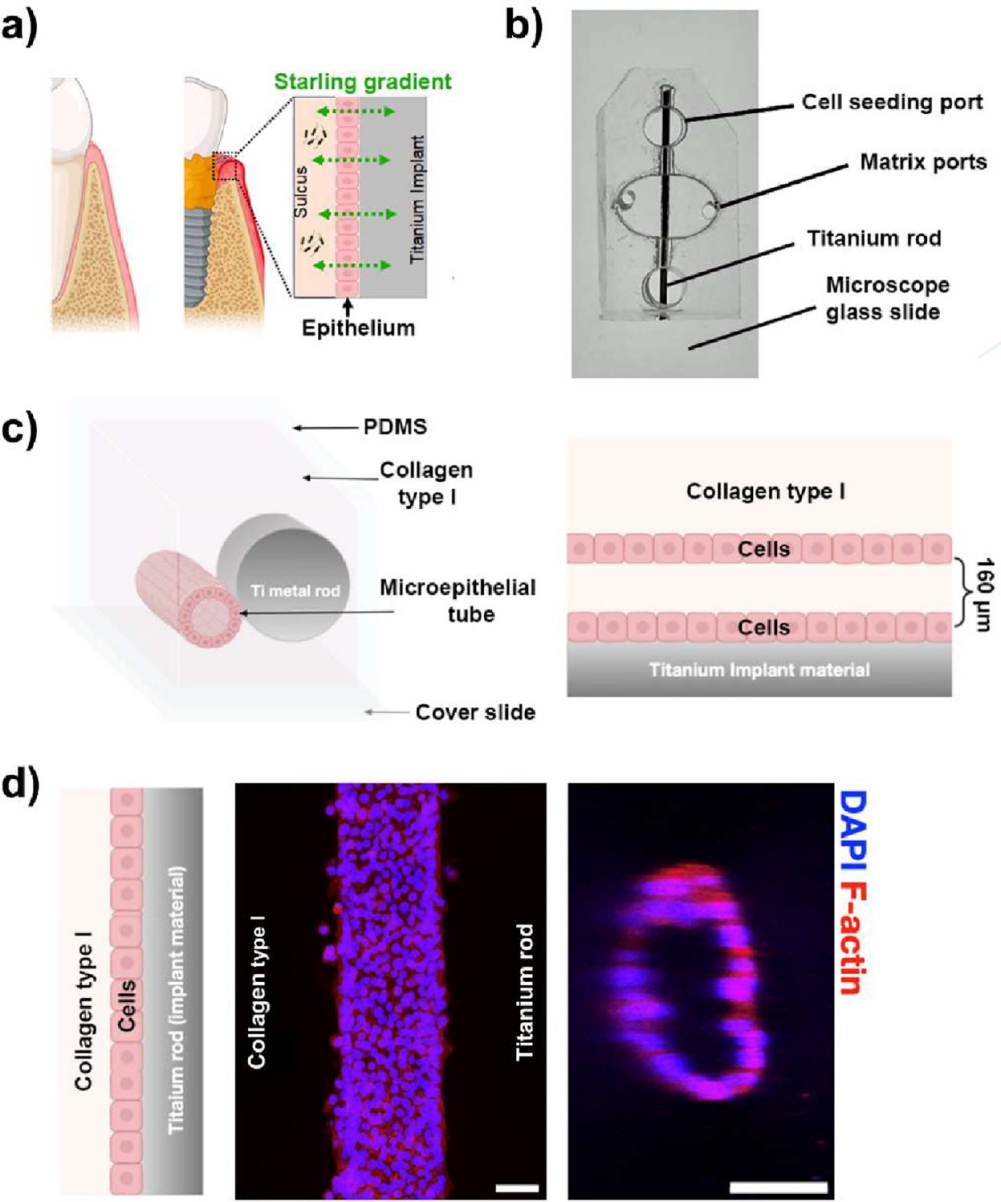
Engineering 3D Peri-implant Epi-mucosa-on-a-chip platform. (a) Schematic illustration comparing healthy peri-implant tissue homeostasis (left) with peri-implantitis condition (right). Increased PICF flow is driven by the starling gradient and associated inflammatory change at the implant-epithelium surface. (b) Photo of the *3D Peri-implant Epi-mucosa-on-a-chip* PDMS device with labeled components. (c) Schematic of the cross-sectional area of the peri-implant mucosa platform. (d) Representative confocal immunofluorescence image illustrating the formation of a microepithelial tube by human immortalized gingival keratinocyte (HIGK) cells between collagen and a titanium metal rod; HIGK cells were stained for F-actin (red) and nuclei with DAPI (blue) (scale bar: 50 μm).

### 3.3 Modification and Characterization of Titanium Metal Roughness

To assess the effect of clinically relevant surface roughness on epithelial barrier integrity, titanium rods were etched with HF for 0–180 s, which leads to acid-etched surfaces with similar parameters to those of commercially available dental implants. SEM revealed the formation of a white honeycomb-like surface morphology after 180 s of etching, demonstrating pronounced topographical alterations (Figure 3a). AFM was subsequently employed to quantify surface roughness (Figure 3b). Compared to untreated samples (0 s), rods etched for 180 s exhibited an approximately 1.5-fold increase in root-mean-square roughness (RMS) and a nearly 2-fold increase in arithmetic average roughness (Ra) (*p* < 0.01). No significant differences in roughness parameters were observed between the 120 and 180 s treatment groups, suggesting a plateau in surface modification beyond 120 s of HF exposure (Figure 3c).

**Figure 3:**
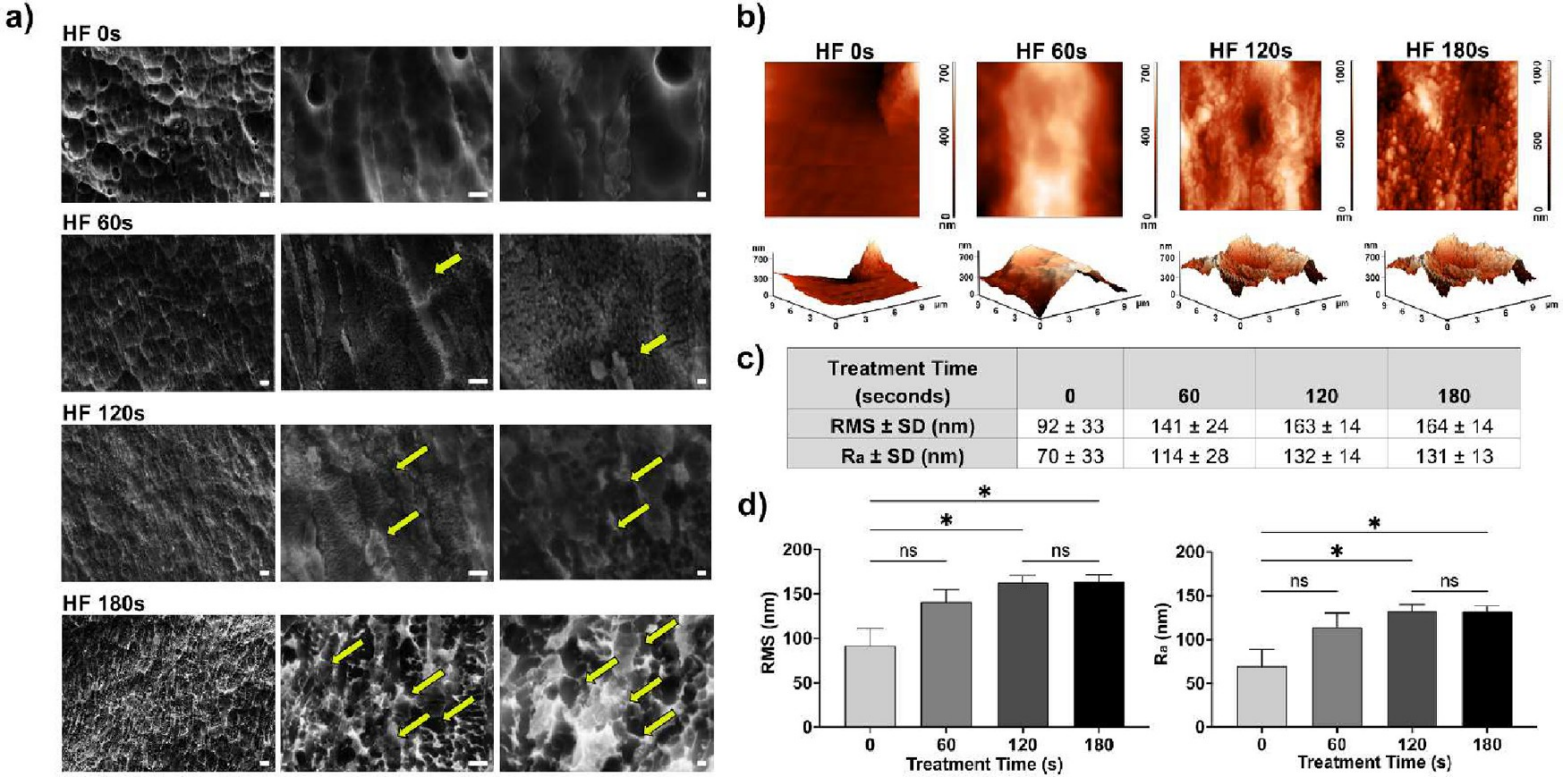
Modification and characterization of titanium metal roughness. (a) Representative scanning electron microscopy (SEM) images of untreated and HF-treated titanium rods following 60, 120, and 180 s of treatment at 2000×, 10,000×, and 20,000× magnifications. Yellow arrows indicate the presence of honeycomb-like microtopography. Scale bar: 2 μm for 2000×; 1 μm for 10,000×; 200 nm for 20,000×. (b) AFM topography images (2D and 3D) of titanium rods acquired over a 5 × 5 μm^2^ scan area with a color scale in nm. (c) Graph depicting surface roughness of 0, 60, 120, and 180 s treated titanium rods, quantified by root-mean-square roughness (RMS) and arithmetic average roughness (Ra) values. (d) Histogram showing the distribution of surface roughness values (RMS and Ra) with standard deviations for untreated (0 s) and 60, 120, and 180 s treated titanium rods. The quantitative data are expressed as means ± SD; *N* = 3, *n* = 3; ns = not significant, *p < 0.05.

### 3.4 Titanium Metal Surface Roughness Affects the Epithelial Barrier

To further evaluate the impact of titanium implants on epithelial barrier function, epithelial cells were cultured in a microfluidic device in the presence of either untreated or HF-treated titanium rods for 180 s. Dextran permeability assays revealed that untreated titanium significantly increased epithelial leakiness by approximately 10-fold (*p* < 0.01) compared with the control condition (Figure 4a,b). Interestingly, HF-treated titanium restored barrier integrity (9.5-fold; *p* < 0.01) compared to untreated rods, suggesting that enhanced surface roughness mitigates epithelial leakiness (Figure 4a,b). This is consistent with the transition from machined to acid-etched titanium surfaces to improve epithelial seal in clinical practice.^50^ Given that epithelial barrier function is regulated by intercellular adhesion molecules such as E-cadherin and tight junction proteins, including ZO-1,^51^ we investigated how the presence of titanium alters the expression and localization of these junctional proteins. Cells lying on untreated titanium exhibited a 5-fold reduction (*p* < 0.0001) in junctional E-cadherin (Figure 4c,d) & (Figure S3) and a 13-fold decrease (*p* < 0.001) in membrane ZO-1 (Figure 4e,f) & (Figure S3) relative to cells cultured without metal, indicating disruption of cell–cell contacts contributing to increased epithelial permeability. Interestingly, cells cultured on HF-treated titanium showed restoration of E-cadherin and ZO-1 localization at cell–cell junctions, indicating that modified microrough titanium surfaces rescued epithelial integrity.

**Figure 4:**
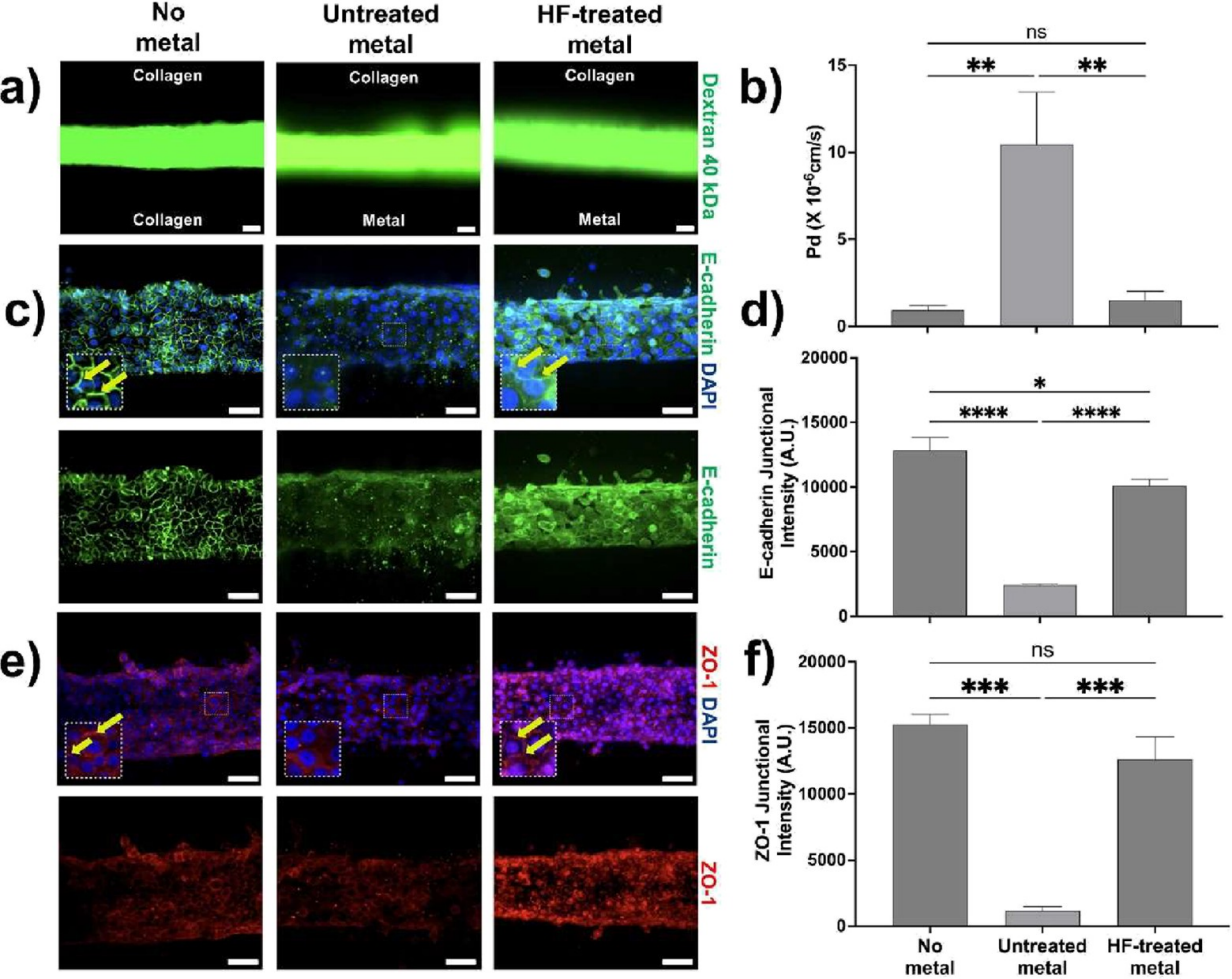
Metal surface roughness alters the epithelial barrier. (a) Representative images of epithelial integrity on *3D Peri-implant Epi-mucosa-on-a-chip* platforms assessed using 40 kDa fluorescently labeled dextran at *t* = 30 s. Conditions include no metal, untreated metal, and HF-treated metal under low mechanical stress (0.1 kPa) (scale bar: 50 μm). (b) Histogram illustrates epithelial leakiness, quantified by the diffusive permeability coefficient (Pd). (c) Representative immunostained images of E-cadherin in epithelium in the absence of metal and in the presence of untreated or treated metal surfaces; HIGKs were stained for DAPI (blue) and E-cadherin (green); yellow arrows indicate examples of membrane-localized E-cadherin (scale bar: 50 μm). (d) Histogram showing E-cadherin junctional (membrane) intensity in arbitrary units (A.U.) as quantified by Junction Mapper. (e) Representative immunostained images of ZO-1 in epithelium in the absence of metal and in the presence of untreated or treated metal surfaces; HIGKs were stained for DAPI (blue) and ZO-1 (red); yellow arrows indicate examples of membrane-localized ZO-1 (scale bar: 50 μm). (f) Histogram illustrating ZO-1 junctional intensity in arbitrary units (A.U.). The quantitative data are expressed as means ± SD; *N* = 3, *n* = 3; ns = not significant, *p < 0.05, **p < 0.01, ***p < 0.001.

### 3.5 High Stress Compromises the Epithelial Barrier

To replicate the mechanical environment of the peri-implant sulcus, which is characterized by the continuous flow of PICF, hydrostatic pressure of 10 kPa (high stress) was applied as previously described to approximate instances of transient increase of crevicular fluid during inflammation.^38,52–56^ Under these high-stress conditions and in the absence of titanium, epithelial permeability increased by approximately 5-fold (*p* < 0.001) (Figure 5a,b), indicating that mechanical stress alone challenges barrier permeability. Additionally, we examined the impact of high hydrostatic pressure on the junctional proteins E-cadherin and ZO-1. Cells exposed to high stress exhibited a 1.7-fold reduction (*p* < 0.05) in junctional E-cadherin (Figure 5c,d) and a 1.6-fold decrease (*p* < 0.05) in membrane-associated ZO-1 (Figure 5e,f) compared to cells cultured without titanium, reflecting the disruption of intercellular adhesion, which increased epithelial permeability. Notably, these changes recapitulate those observed in human tissues during peri-implantitis inflammation in Figure 1, thus providing validation of the translational relevance of the microfluidic systems model and its sensitivity to capture biological changes in response to local stress. Even under high-stress conditions, cells cultured on HF-treated titanium showed restored localization of E-cadherin (Figure 5c,d) and ZO-1 (Figure 5e,f) at the cell membrane, indicating maintenance of epithelial barrier integrity on a titanium microrough substrate similar to peri-implant health (Figure 5c–f). These findings collectively demonstrate that both surface roughness and mechanical stress critically influence epithelial barrier integrity, with HF-induced surface modification effectively mitigating implant-associated barrier compromise under both physiological and elevated stress conditions, suggesting that modified microrough implant surfaces can maintain an epithelial seal in physiological conditions in the absence of inflammation.

**Figure 5:**
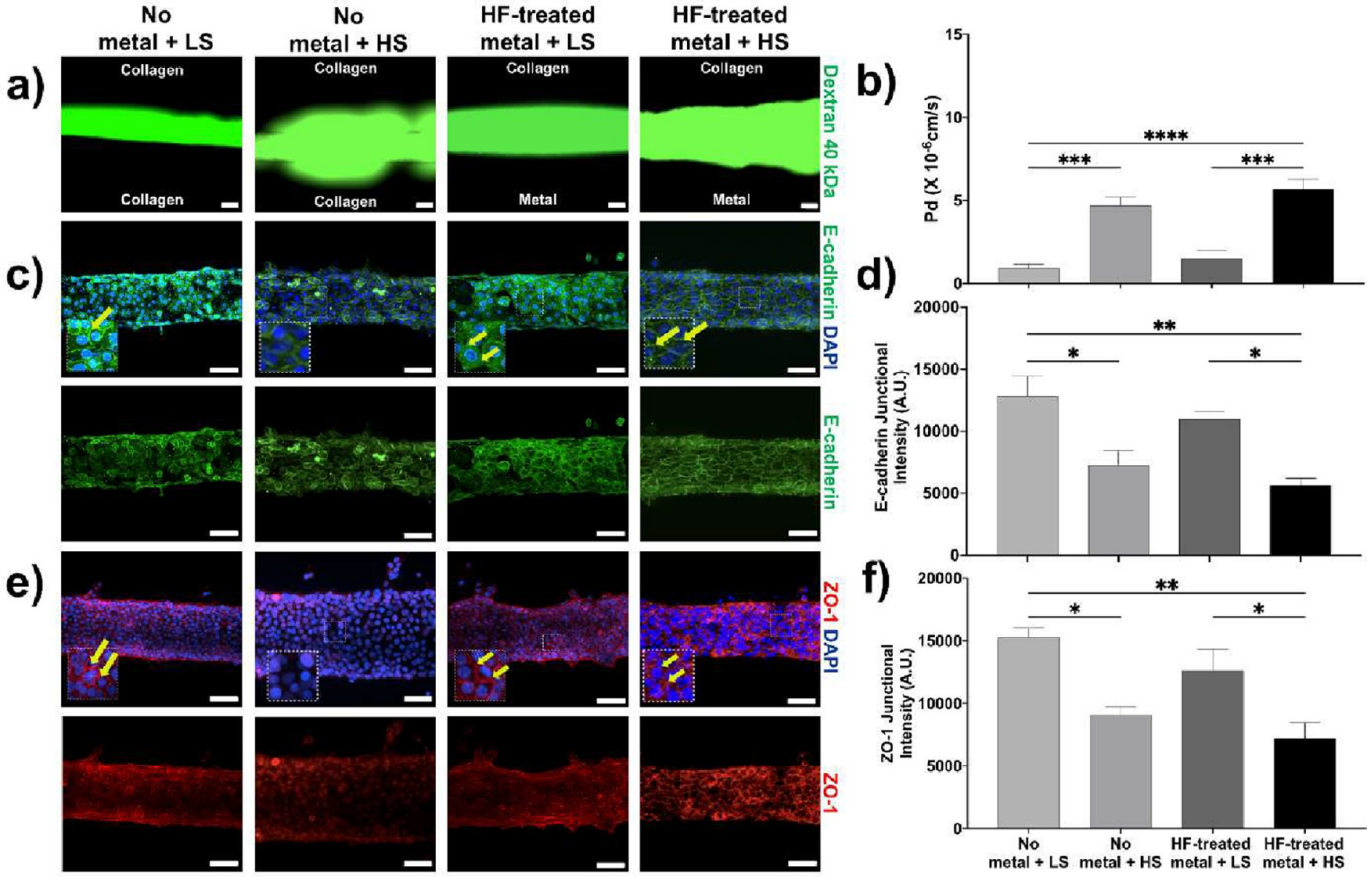
High stress compromises the epithelial barrier integrity. (a) Representative fluorescence images showing epithelial barrier integrity on *3D Peri-implant Epi-mucosa-on-a-chip* platforms, assessed by the diffusion of 40 kDa fluorescently labeled dextran at *t* = 30 s. Conditions include no metal and HF-treated metal, each under low stress (LS; 0.1 kPa) and high stress (HS; 10 kPa) (scale bar: 50 μm). (b) Histograms illustrate epithelial leakiness, quantified by the diffusive permeability coefficient (Pd). (c) Representative immunofluorescence images showing epithelial intercellular interactions via E-cadherin staining under high-stress (HS) and low-stress (LS) conditions, both without metal and in the presence of HF-treated metal; HIGKs were stained for DAPI (blue) and E-cadherin (green); yellow arrows indicate examples of membrane-localized E-cadherin (scale bar: 50 μm). (d) Histogram showing E-cadherin junctional intensity in arbitrary units (A.U.) as quantified by Junction Mapper. (e) Immunofluorescence staining of ZO-1 in HIGKs cultured on 3D peri-implant Epi-Mucosa-on-a-Chip platforms under high-stress (HS) and low-stress (LS) conditions, both without metal and in the presence of HF-treated metal; HIGKs were stained for DAPI (blue) and ZO-1 (red); yellow arrows indicate examples of membrane-localized ZO-1 (scale bar: 50 μm). (f) Histogram illustrating ZO-1 junctional intensity quantified using Junction Mapper and expressed in arbitrary units (A.U.). The quantitative data are expressed as means ± SD; *N* = 3, *n* = 3; ns = not significant, *p < 0.05, **p < 0.01, ***p < 0.001, ****p < 0.0001.

### 3.6 Bacterial Challenge Increases Epithelial Leakiness

Next, we extended our investigation to evaluate the effect that peri-implant bacterial challenge has on epithelial barrier integrity. *P. gingivalis*, a Gram-negative anaerobic pathogen implicated in peri-implantitis^17,57–59^ was introduced in the model at MOI = 50 for 15 min. Upon *P. gingivalis* exposure, the cells demonstrated a significant increase in epithelial leakiness by 5-fold (*p* < 0.05), compared to nonchallenged cells (Figure 6a,b). Notably, the impact of *P. gingivalis* was further amplified under HF-treated titanium conditions (Figure 6a,b) and untreated conditions (Figure S4a,b), indicating the negative impact of the bacterial-driven inflammation to the epithelial barrier. Interestingly, cells cultured on HF-treated titanium exhibited significantly reduced leakiness compared to untreated metal (4.8-fold; *p* < 0.01) even in the presence of *P. gingivalis*, indicating that the peri-implant microenvironment via modulations of surface roughness plays a dominant role in preserving epithelial integrity relative to bacterial challenge alone (Figure S4a,b). To further elucidate the mechanisms underlying the observed increase in permeability, we investigated the membrane localization of E-cadherin and ZO-1. Immunofluorescence and quantitative analysis revealed a marked reduction in membrane-associated E-cadherin and ZO-1 in cells exposed to *P. gingivalis*, with approximately 3-fold (*p* < 0.001) and 1.8-fold (*p* < 0.01) decreases, respectively (Figure 6c–f). This loss of junctional proteins likely accounts for the increased leakiness observed and reflects disruption of epithelial adhesion and is aligned with the same patterns noted in human peri-implant inflammatory lesions as compared to health (Figure 1). Furthermore, even under HF-treated titanium conditions, exposure to *P. gingivalis* resulted in a significant reduction of E-cadherin and ZO-1 localization at cell–cell junctions by approximately 1.9-fold (*p* < 0.01) and 2.46-fold (*p* < 0.01), respectively compared to no-exposed cells (Figure 6c–f). Interestingly, under high stress conditions, exposure to *P. gingivalis* on HF-treated titanium resulted in a significant increase of junctional E-cadherin and ZO-1 by approximately 5-fold (*p* < 0.0001) and 5.3-fold (*p* < 0.0001), respectively, compared to *P. gingivalis*-exposed cells on untreated titanium (Figure S4c,d). Overall, these findings further support that implant surface properties significantly influence host barrier responses, even under pathogenic and mechanically stressed conditions.

**Figure 6:**
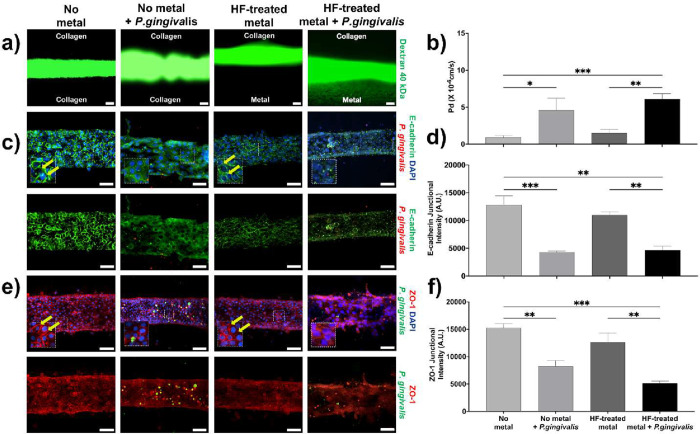
*P. gingivalis*increases the epithelial leakiness. (a) Representative images assessing epithelial integrity on 3D peri-implant mucosa platforms. Integrity was evaluated by permeability to fluorescently labeled 40 kDa dextran at *t* = 30 s. Conditions include no metal control vs HF-treated metals, with or without *P. gingivalis* exposure (MOI = 50), under low (0.1 kPa) or high (10 kPa) mechanical stress (scale bar: 50 μm). (b) Histograms illustrating epithelial leakiness, quantified by diffusive permeability coefficient (*P*_d_). (**c**) Immunofluorescence staining of E-cadherin in HIGKs cultured on *3D Peri-implant Epi-Mucosa-on-a-Chip* platforms, with or without *P. gingivalis* exposure (MOI = 50), in the absence of metal and in the presence of HF-treated metal; HIGKs were stained for DAPI (blue), E-cadherin (green), and *P. gingivalis* (red); yellow arrows indicate examples of membrane-localized E-cadherin (scale bar: 50 μm). (d) Histogram showing E-cadherin junctional intensity in arbitrary units (A.U.). (e) Immunofluorescence staining of ZO-1 in HIGKs cultured on *3D peri-implant Epi-Mucosa-on-a-Chip* platforms, with or without *P. gingivalis* exposure (MOI = 50), in the absence of metal and in the presence of HF-treated metal; HIGKs were stained for DAPI (blue), ZO-1 (red), and *P. gingivalis* (green); yellow arrows indicate examples of membrane-localized ZO-1 (scale bar: 50 μm). (f) Histogram illustrating ZO-1 junctional intensity expressed in arbitrary units (A.U.). The quantitative data are expressed as means ± SD; *N* = 3, *n* = 3; ns = not significant, *p < 0.05, **p < 0.01, ***p < 0.001.

### 3.7 Titanium Particles Increase Epithelial Leakiness

Finally, we investigated the effects of i-TiPs, which are consistently found in the majority of peri-implantitis cases and are associated with microbial dysbiosis and host immune pro-inflammatory effects that modify response to bacteria. Therefore, i-TiPs are a critical disease modifier participating in the inflammatory process in peri-implantitis.^21^ Based on in vivo data^22^ reflecting local concentration gradients in humans, i-TiPs were embedded in the collagen matrix of the microfluidic devices at a concentration of 10^4^ particles/mL. SEM analysis (Figure 7a) revealed that i-TiPs significantly altered the matrix structure, leading to enhanced porosity (Figure 7b), increased fiber diameter (Figure 7c), and the formation of bundled structures. These findings suggest that i-TiPs substantially modify the extracellular matrix environment in which the cells reside. Moreover, mechanical characterization via shear modulus measurements showed that the presence of i-TiPs increased the matrix stiffness by approximately 2.4-fold (*p* < 0.05) compared to matrices without i-TiPs (Figure 7d,e), further indicating a mechanical alteration of the cellular substrate. To assess functional consequences, we evaluated epithelial barrier integrity using a dextran permeability assay (Figure 8a,b). Cells cultured on collagen I matrices containing i-TiPs exhibited approximately 3-fold (*p* < 0.01) greater epithelial leakiness compared to cells grown on HF-treated metal surfaces. The addition of *P. gingivalis* further increased leakiness by 9-fold (*p* < 0.0001), suggesting a synergistic effect of bacterial challenge and i-TiPs on barrier disruption (Figure 8a,b). These functional impairments corresponded with molecular changes in junctional proteins. Specifically, i-TiPs reduced membrane-associated levels of E-cadherin (Figure 8c,d) and ZO-1 (Figure 8e,f) by 1.2-fold and 1.3-fold, respectively, compared to HF-treated metal. The presence of *P. gingivalis* exacerbated this effect, decreasing E-cadherin (Figure 8c,d) and ZO-1 (Figure 8e,f) levels by an additional 8-fold and 6-fold, respectively. These results collectively demonstrate that i-TiPs compromise the structural and mechanical integrity of the extracellular matrix and weaken epithelial barrier function, a process further aggravated by bacterial presence.

**Figure 7:**
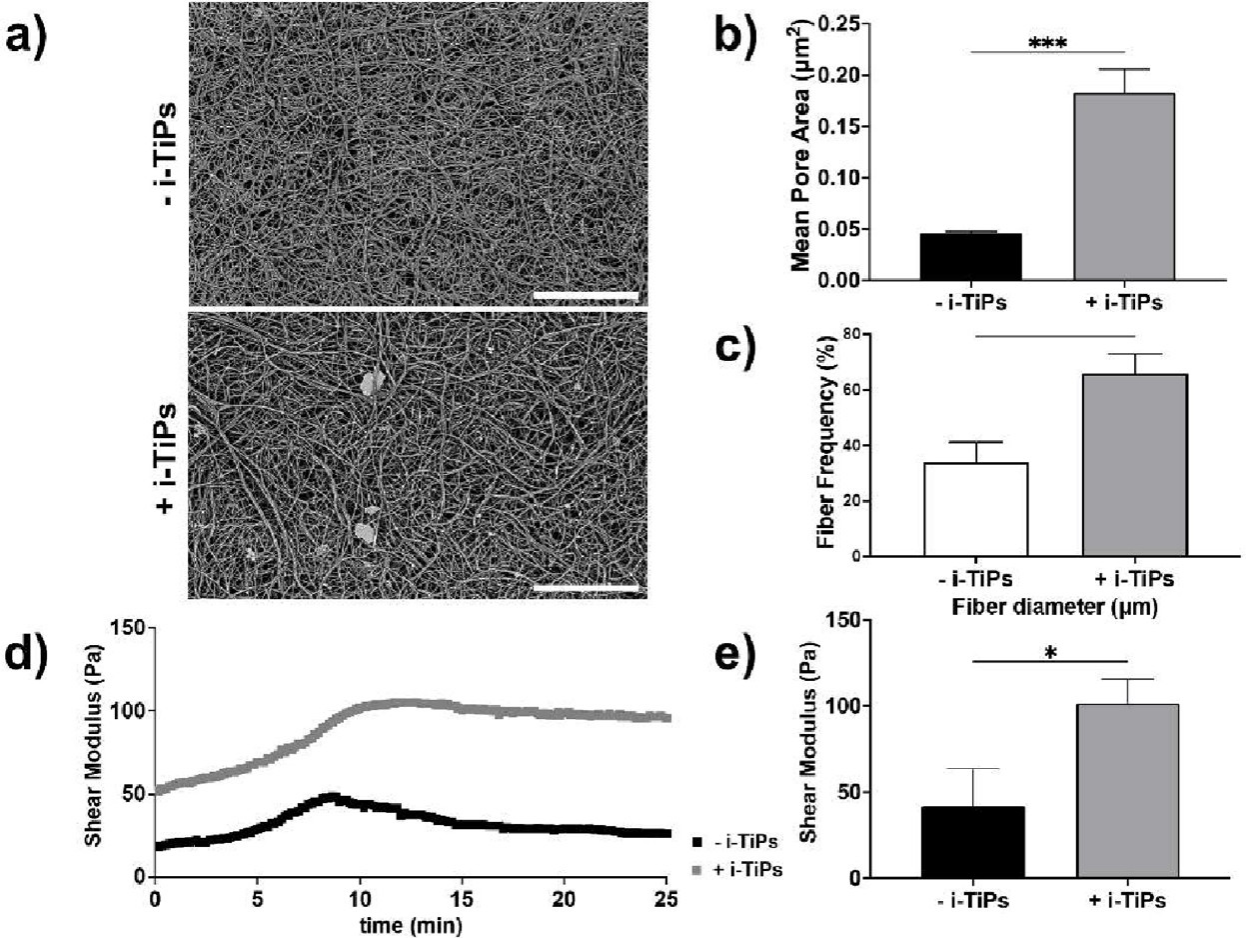
Implant-derived titanium particles (i-TiPs) modify the extracellular matrix environment. (a) Representative scanning electron microscopy (SEM) images of type I collagen matrices with (+i-TiPs) and without (−i-TiPs) incorporated i-TiPs. (scale bar: 10 μm). (b) Histogram analysis of mean pore area (μm^2^) for collagen I matrices with and without i-TiPs. (c) Histogram analysis of fiber diameter distribution (*d* > 120 nm and *d* < 120 nm) collagen I matrices with and without i-TiPs. (d) Rheological measurement of shear modulus (Pa) over a 25 min period. (e) Shear modulus (Pa) at 25 min for −i-TiPs and +i-TiPs conditions. (f) Quantification of shear modulus (Pa) at 25 min for −i-TiPs and +i-TiPs conditions, presented as mean ± SD; *N* = 3, *n* = 3. *p < 0.05, ***p < 0.001.

**Figure 8:**
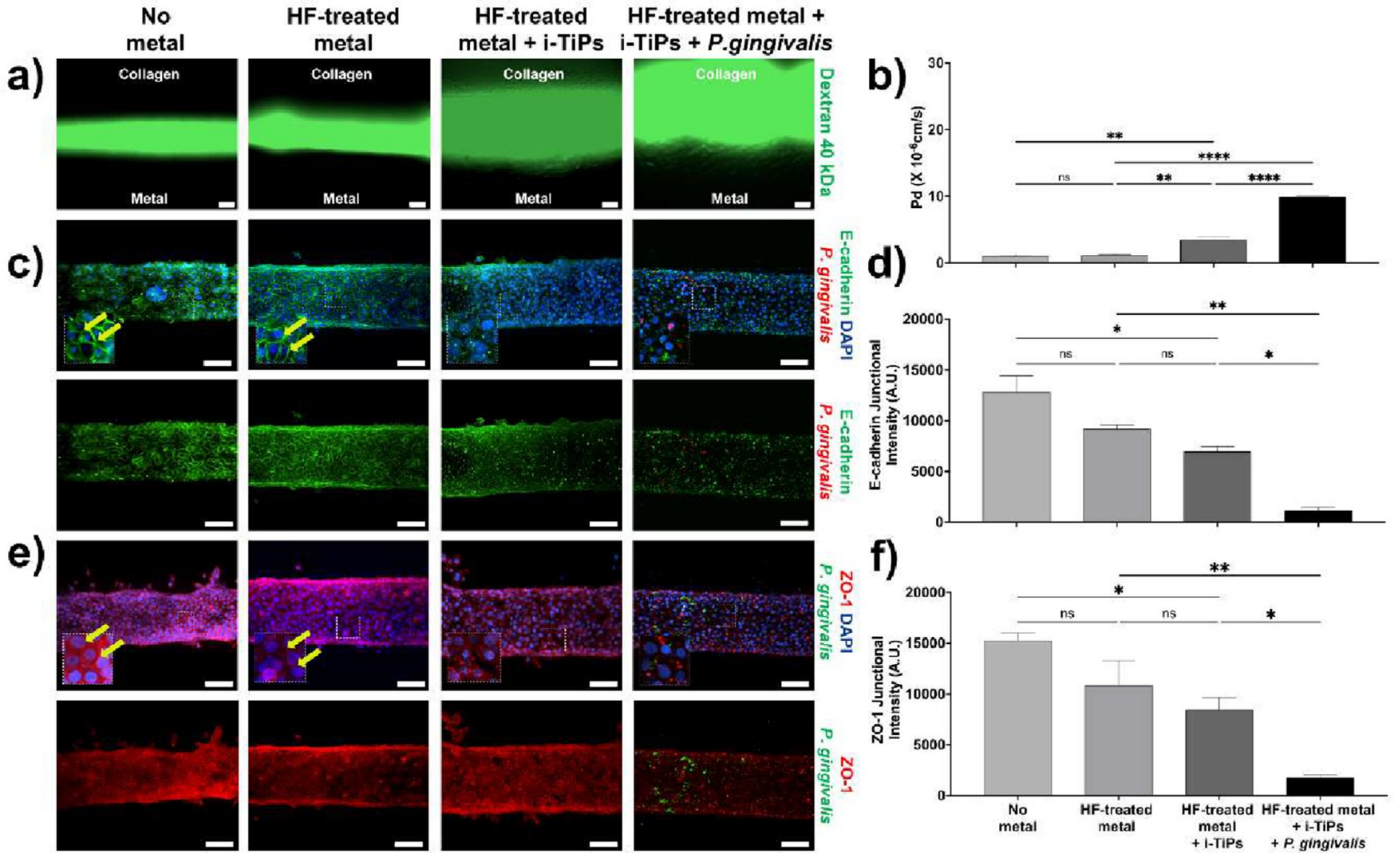
Implant-derived titanium particles increase the epithelial leakiness. (a) Representative images showing epithelial integrity on *3D Peri-implant Epi-mucosa-on-a-chip* Platforms, assessed by diffusion of 40 kDa fluorescently labeled dextran at 30 s. Conditions include no metal (control), HF-treated metal, HF-treated metal with i-TiPs, and HF-treated metal with i-TiPs and *P. gingivalis* exposure (MOI = 50) (scale bar: 50 μm). (b) Quantification of dextran permeability expressed as the diffusive permeability coefficient (Pd). (c) Representative immunofluorescence images showing epithelial intercellular interactions via E-cadherin staining under no metal, HF-treated metal, HF-treated metal with i-TiPs, and HF-treated metal in the presence of i-TiPs and low-stress (LS) conditions, both without metal and in the presence of HF-treated metal and *P. gingivalis*. HIGKS were immunostained for E-cadherin (green) and DAPI (blue), with *P. gingivalis* (red) where applicable; yellow arrows indicate examples of membrane-localized E-cadherin (scale bar: 50 μm). (d) Quantification of E-cadherin junctional intensity in arbitrary units (A.U.). (e) Representative immunofluorescence images showing epithelial intercellular interactions via ZO-1 staining under no metal, HF-treated metal, HF-treated metal with i-TiPs, and HF-treated metal in the presence of i-TiPs and low-stress (LS) conditions, both without metal and in the presence of HF-treated metal and *P. gingivalis*. HIGKs were immunostained for ZO-1 (red) and DAPI (blue), with *P. gingivalis* (green) where applicable; yellow arrows indicate examples of membrane-localized ZO-1 (scale bar: 50 μm). (f) Quantification of ZO-1 junctional intensity expressed in arbitrary units (A.U.). The quantitative data are expressed as means ± SD; *N* = 3, *n* = 3; ns = not significant, *p < 0.05, **p < 0.01, ***p < 0.001, ****p < 0.0001.

## 4 Discussion

The structural and functional integrity of the peri-implant epithelium is critical for maintaining a protective seal against the microbial-rich oral environment.^12,60,61^ In this study, we integrated clinical tissue analysis with a novel *3D Peri-implant Epi-mucosa-on-a-chip* platform to dissect how implant characteristics, mechanical forces, microbial challenges, and implant debris collectively contribute to epithelial barrier dysfunction in peri-implantitis. Our findings reveal that epithelial barrier breakdown in peri-implantitis is associated with morphological disruption, increased intercellular edema, and diminished expression of junctional proteins E-cadherin and ZO-1. Importantly, this breakdown is not solely a biological response but is strongly modulated by the material properties of the implant, such as the surface topography and the presence of titanium particles.

Histological evaluation of human gingival tissues revealed that peri-implantitis tissues exhibit significant architectural disruption compared to healthy and periodontally healthy tissues. Specifically, similar to previous studies, we observed irregular rete ridges, severe intercellular edema, and dense inflammatory infiltrates within both the junctional and sulcular epithelium.^62–64^ Cell adhesion molecules, notably E-cadherin and ZO-1, are essential for maintaining epithelial barrier integrity and oral tissue homeostasis. Therefore, disruption in the regulation of these adhesion molecules has been implicated in the onset and progression of various inflammatory diseases, where compromised epithelial barriers facilitate microbial invasion and perpetuate chronic inflammation.^38,51,65,66^ Immunofluorescence analyses further confirmed that E-cadherin and ZO-1 were either weakly expressed or absent in peri-implantitis tissues. These findings underscore a critical link between structural compromise and molecular dysregulation of the epithelial barrier in peri-implantitis.

A comprehensive understanding of oral pathophysiology in peri-implantitis necessitates biomimetic 3D culture systems capable of recapitulating the extracellular matrix (ECM) architecture, mechanical environment, and dynamic cell–cell and cell–matrix interactions observed in vivo.^38,65–67^ Traditional 2D models and animal studies often fall short in capturing this complexity, limiting mechanistic insight and translational potential. Recent advances in organ-on-a-chip technologies have enabled accurate epithelial stratification and differentiation,^67–71^ effectively mimicking gingival function in both healthy and diseased states.^67,70–73^ Examples include the Gingival Crevice-on-a-Chip, which models host–microbiome interactions,^72,74^ the oral mucosa-on-a-chip for studying microbial responses to dental materials,^75^ the humanized model of the periodontal gingival pocket for examining host–pathogen imbalances and inflammatory responses,^69^ and oral mucositis-on-a-chip, designed to evaluate damage induced by chemo- and radiotherapy.^76^ While Lee et al. developed a *3D Oral Epi-mucosa-on-a-chip* platform to replicate the mechanical microenvironment of oral epithelium by investigating the role of dynamic hydrostatic stress, ECM stiffness, and composition in epithelial barrier.^38^ Herein, we aimed to mechanistically investigate the impact of material and environmental factors on peri-implant epithelial integrity, we developed a micro engineered *3D Peri-Implant Epi-mucosa-on-a-chip* system. This platform enables the investigation of key features of the oral peri-implant epithelial barrier, including tunable implant surface properties, the presence of bacteria, and biomechanical forces.

Using this system, we demonstrated that titanium surface roughness exerts a significant effect on epithelial function. Specifically, HF-etched titanium, characterized by a distinct honeycomb-like microtopography, preserved epithelial adhesion and junctional protein localization. In contrast, smooth, untreated titanium induced epithelial leakiness and disrupted E-cadherin and ZO-1 distribution. These findings align with previous findings suggesting that moderate surface roughness enhances cell viability, adhesion and intercellular organization by promoting favorable cell–material interactions.^40,77–81^

While biological mechanisms play a key role in regulating barrier function, mechanical forces are also significant contributors. In the oral mucosa, interstitial forces, driven by hydrostatic pressure, maintain tissue homeostasis. Specifically, interstitial and mechanical forces contribute positively to tissue development and epithelial barrier function tissue homeostasis.^38,56,70,82^ To this end, masticatory loading induces interstitial fluid flow within the periodontal ligament (PDL) and alveolar bone, generating fluid shear stress that regulates cytoskeletal organization, modulates tissue remodeling, and shapes the immunoregulatory and barrier-protective responses of interstitial/mechanical forces in sustaining tissue homeostasis.^82,83^ Specifically, under healthy conditions, interstitial pressure typically ranges from 0.2 to 2 kPa.^54–56^ In our experiment, the selected hydrostatic pressure values of 0.1 and 10 kPa represent physiologically relevant ranges to simulate baseline interstitial pressure and elevated inflammatory mechanical stress, respectively.^52–56^ The lower value approximates homeostatic interstitial fluid pressure commonly measured in periodontal tissues, while the higher value reflects increased pressure conditions observed during inflammation, consistent with ranges reported in experimental models of periodontal ligament stress and connective tissue inflammation.^52–56^ Experimental application of mechanical stress simulating PICF pressure further impaired barrier function, supporting the concept that biomechanical forces in vivo along with material cues to destabilize the epithelial seal. The presence of high mechanical stress generated by the application of high pressure of 10 kPa, diminished barrier integrity and worsened junctional organization, emphasizing the synergistic impact of mechanical forces and material properties on epithelial stability. Importantly, surfaces treated with HF mitigated these negative effects, suggesting that HF-treated titanium may enhance epithelial resilience under mechanical stress and serve as a promising surface modification strategy.

In addition, we examined the impact of pathogenic challenges with *P. gingivalis*, a key bacteria population involved in peri-implantitis.^17,58,59^ To assess early host responses to acute bacterial challenge, cells were exposed to *P. gingivalis* at an MOI of 50 for 15 min under aerobic conditions. Although *P. gingivalis* is an anaerobe, previous studies have demonstrated that it exhibits considerable aerotolerance, allowing the bacterial population to remain viable during brief oxygen exposure (e.g., 15 min).^43–45^ Nakayama et al. reported that this aerotolerance is mediated by superoxide dismutase (SOD), which catalyzes the disproportionation of superoxide radicals generated during partial oxygen reduction, enabling bacterial viability to be maintained for at least 5 h under aerobic conditions.^44^ However, extended aerobic incubation markedly reduces survival and proliferation.^43,44,84^ Future studies incorporating prolonged infection models under anaerobic conditions will be necessary to better capture the dynamics of sustained *P. gingivalis*–epithelium interactions.

Our results demonstrated that *P. gingivalis* is responsible for the increase in epithelial leakiness and disruption of intercellular adhesion consistent with previous studies.^13,57^ While cells cultured on untreated titanium exhibited severe junctional disruption and increased leakiness upon *P. gingivalis* exposure, those on HF-treated titanium maintained significantly better barrier integrity. In addition, junctional E-cadherin and ZO-1 remained localized at the cell membrane, and permeability assays showed reduced leakiness relative to the untreated titanium group. These results suggest that HF-treated titanium surfaces confer a protective effect against microbial-induced epithelial compromise. This may be attributed to enhanced epithelial adhesion and stabilization of junctional complexes facilitated by the modified surface topography. Thus, beyond their mechanical and structural influence, surface-engineered implants appear to reinforce epithelial resilience in the presence of pathogenic bacteria.^85^

Finally, we investigated the impact of i-TiPs, which, due to mechanical wear, corrosion, or surgical manipulation, accumulate in peri-implant tissues and are increasingly recognized not only as byproducts but as active mediators of peri-implantitis.^20–23,25–29^ To mimic the in vivo presence of i-TiPs, we incorporated them into the collagen matrix of our *3D Peri-implant Epi-mucosa-on-a-chip* platform. Structural analysis revealed that the introduction of i-TiPs significantly disrupted matrix microstructure. Specifically, we observed increased collagen fiber thickness, greater porosity, and abnormal fiber bundling, all of which contribute to an altered mechanical environment. Mechanical testing further demonstrated that these changes resulted in a marked increase in matrix stiffness, suggesting that i-TiPs alter the biophysical properties of the cellular substrate in a way that could mediate epithelial leakiness.^38^ Specifically, the presence of i-TiPs led to a significant increase in epithelial leakiness which was further exacerbated in the presence of *P. gingivalis*, highlighting a synergistic interaction between biomechanical stressors and microbial insult. The observed increase in permeability was correlated with intercellular adhesion integrity as driven by the substantial reductions in membrane-localized E-cadherin and ZO-1 in epithelial cells exposed to i-TiPs. Taken together, these results provide strong evidence that i-TiPs serve as both structural and functional disruptors of the peri-implant epithelial barrier.

To summarize, our study provides critical mechanistic insight into the multifactorial nature of epithelial barrier failure in peri-implantitis. It highlights the complex interplay between surface topography, mechanical stress, microbial challenge, and implant-derived particles in modulating epithelial stability. Importantly, we demonstrate that surface modifications, specifically HF-etching, can effectively restore barrier integrity across a range of pathological insults. These findings significantly advance our understanding of peri-implant disease pathogenesis and highlight the potential of surface-engineered implants as a promising therapeutic strategy to enhance soft tissue integration and minimize disease recurrence. Given that the peri-implant and periodontal mucosa consists of a stratified epithelial layer, future work will build upon the current model to further refine the epithelial architecture and achieve a stratified and differentiated structure incorporating additional cell populations, such as parenchymal cells.^67–69^ In parallel, efforts will be directed toward incorporating immune components, such as neutrophils and macrophages, to better capture the complex inflammatory and remodeling dynamics that occur in vivo.^86–88^ The integration of these cellular and structural elements will enable a more comprehensive representation of the host–implant interface, providing a powerful platform to study the cellular crosstalk and signaling mechanisms underlying tissue integration, inflammation, and barrier dysfunction. Ultimately, these advancements will contribute to the development of improved therapeutic and biomaterial strategies aimed at promoting long-term peri-implant health and stability.

## Supplementary Material





## Data Availability

The data that support the findings of this study are available from the corresponding author upon reasonable request.

## Notes

Representative time-lapse videos showing diffusion of 40 kDa dextran for permeability (*P*_d_) analysis (MP4)
Schematic workflow of the 3D Peri-implant Epi-mucosa-on-a-chip platform; titanium particle analysis in human tissues; negative control staining with isotype-matched IgG; and epithelial barrier integrity compromised under high mechanical stress and in the presence of untreated metal (PDF)
