# Supplementary material for: 3D Peri-Implant Epi-Mucosa-on-a-Chip Reveals Alterations in Epithelial Barrier Function Mediated by Host-Bacteria-Biomaterial Interactions
Source: ACS Biomater Sci Eng. 2025 Nov 12;11(12):7134–48. doi: 10.1021/acsbiomaterials.5c01232 (PMC12690518; doi:10.1021/acsbiomaterials.5c01232)
Supplement: Supplementary file 2 [file ab5c01232_si_002.pdf]

Supporting Information

Title: 3D Peri-implant Epi-mucosa-on-a-chip reveals alterations in epithelial barrier function mediated by host-bacteria-biomaterial interactions

Sana Surrency[1,2#], Soraya Tarrah[1,2#,] Mashael Al Thuanayan[3], Yoontae Kim[1,2], Rahul Patil[1,2], Alison Grafton[1,2], Micaila Curtis[1,2], Peter Lialios[1,2], Georgios A. Kotsakis[4], Stella Alimperti[1,2,5*]

[1]Department of Biochemistry and Molecular & Cellular Biology, Georgetown University, Washington, DC 20057, USA.

[2]Center for Biological and Biomedical Engineering, Georgetown University, Washington, DC 20057, USA.

[3]King Abdullah bin Abdulaziz University Hospital, Riyadh 11564, Saudi Arabia.

[4]Department of Oral Biology, Rutgers School of Dental Medicine, Newark, NJ 07103, USA.

[5]Institute for Soft Matter Synthesis and Metrology, Georgetown University, Washington, DC 20057, USA.

Figure S1

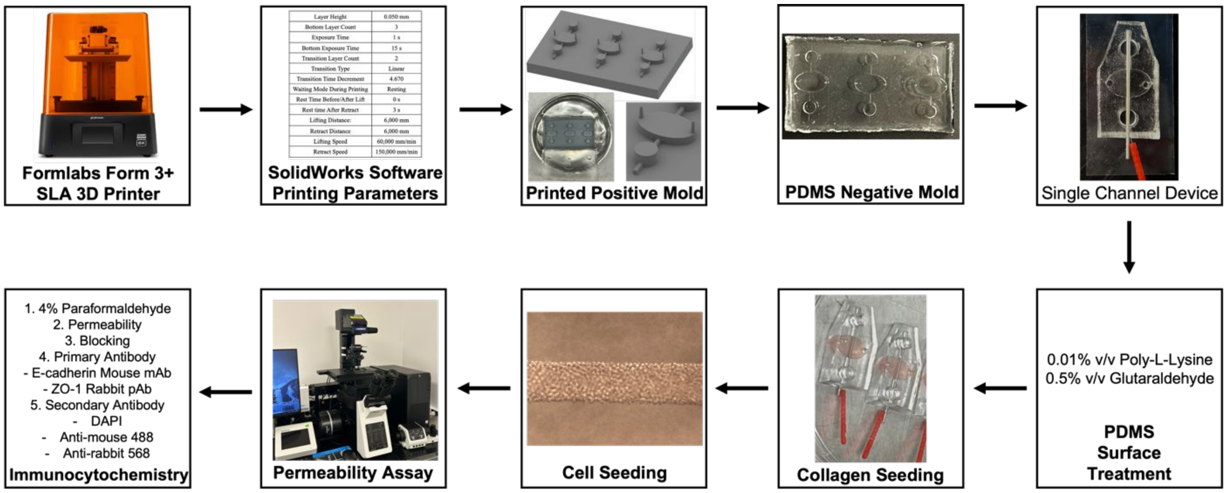

Figure S1. Schematic workflow of the 3D Peri-implant Epi-mucosa-on-a-chip platform.

Figure S2

a) b)

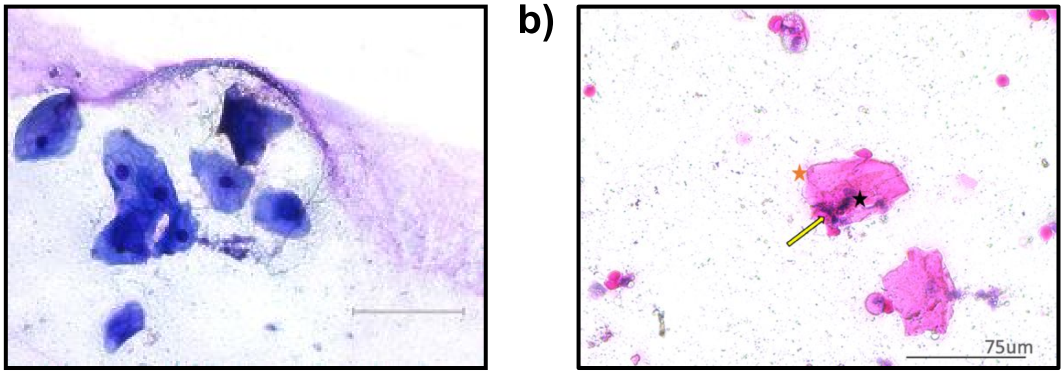

Figure S2. Titanium particle analysis in human tissues. (a) Smear sample of peri-implant healthy tissue. Epithelial cell sample is negative of titanium particles. (b) Smear sample of peri-implantitis tissue with an epithelial cell (orange star) and nucleus (black star). Sample is positive for sub-micron titanium particles in the cytosol (arrow). (Scale bar: 75 μm).

Figure S3

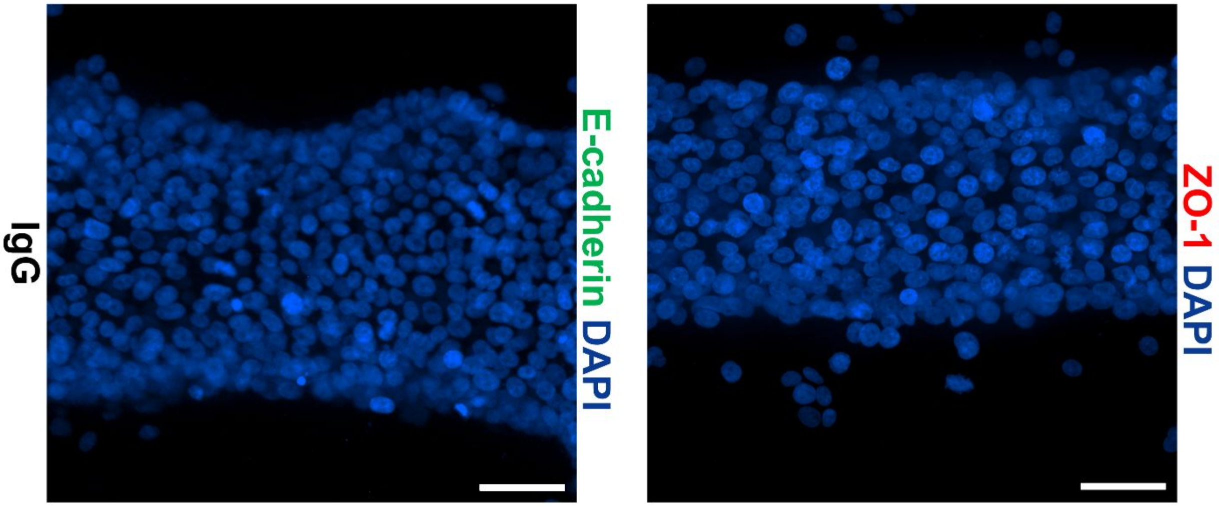

Figure S3. Negative control staining with isotype-matched IgG in HIGKs. Nuclei counterstained with DAPI (blue); E-cadherin (green); ZO-1 (red); Scale bar: 50 µm.

Figure S4

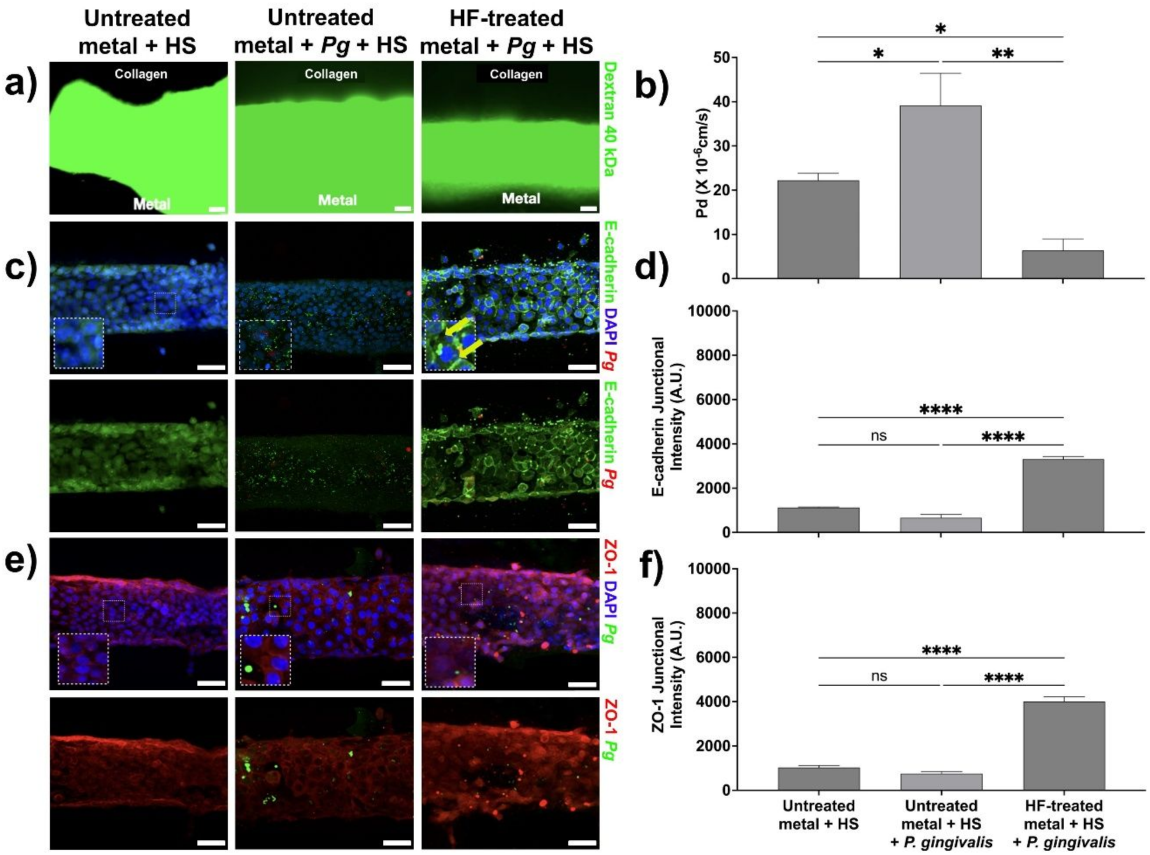

Figure S4. Epithelial barrier integrity is compromised under high mechanical stress and in the presence of untreated metal

(**a**) Representative images showing epithelial integrity in 3D Peri-implant Epi-mucosa-on-a-chip platforms, assessed by diffusion of 40 kDa fluorescently labeled dextran at 30 seconds. Conditions include untreated metal, untreated metal with *P. gingivalis* exposure (MOI = 50), and HF-treated metal with *P. gingivalis* exposure (MOI = 50), all under high mechanical stress (10 kPa) (scale bar: 50 µm). (**b**) Quantification of dextran permeability expressed as the diffusive permeability coefficient (P_d). (**c**) Representative maximum projection confocal images of epithelium, immunostained for E-cadherin (green) and DAPI (blue), with *P. gingivalis* (red) where applicable; yellow arrows indicate examples of membrane-localized E-cadherin (Scale bar: 50 µm). (**d**) Quantification of E-cadherin junctional intensity expressed in arbitrary units (A.U.). (**e**) Representative maximum projection confocal images of epithelium, immunostained for ZO-1 (red) and DAPI (blue), with *P. gingivalis (*green) where applicable; yellow arrows indicate examples of

membrane-localized ZO-1 (Scale bar: 50 μm). (**f**) Quantification of ZO-1 junctional intensity expressed in arbitrary units (A.U.). The quantitative data are expressed as means ± SD; N = 3, n = 3; ns = not significant, *, $p < 0.05$, **, $p < 0.01$, ***, $p < 0.001$.

Supplementary Video S1. Representative time-lapse videos showing diffusion of 40 kDa dextran for permeability (P_d) analysis. The videos illustrate dextran diffusion into the collagen interstitial matrix under low mechanical stress (0.1kpa) for (a) no metal and (b) metal conditions.
